# Perirectal Extragastrointestinal Stromal Tumor: An Unusual Presentation

**DOI:** 10.7759/cureus.15529

**Published:** 2021-06-08

**Authors:** Mohamed M Elagami, Alman Khalid, Vinod Kumar, Monisha Singhal, Matthew A Grossman

**Affiliations:** 1 Internal Medicine, St. Joseph's University Medical Center, Paterson, USA; 2 Hematology/Oncology, St. Joseph's University Medical Center, Paterson, USA; 3 Interventional Gastroenterology, St. Joseph's University Medical Center, Paterson, USA

**Keywords:** gist, egist, cells of cajal, multidisciplinary, endoscopic, imatinib mesylate

## Abstract

Gastrointestinal stromal tumors (GISTs), even though rare, remain the most common mesenchymal tumors of the gastrointestinal (GI) tract. When GISTs occur outside of the GI tract, they are termed extragastrointestinal stromal tumors (EGISTs). Most GISTs arise from the stomach (50-70%) and small intestine (20-30%). A smaller percentage of these tumors also occurs in the large intestine (5%) and esophagus (2-5%). EGISTs have histopathological and molecular characteristics that are similar to GISTs. However, the precise incidence and tumor behavior of EGISTs are not fully understood. EGISTs have no specific symptoms or radiologic features, and in most cases, the presenting complaint is abdominal pain or discomfort. Yet, they tend to be more aggressive and have a worse prognosis than GISTs. Morphologic diagnosis based on microscopic examination of histological sections is the standard diagnostic procedure for GIST/EGIST. In this patient-centered study, we present a case of EGIST that originated in the anterior perirectal space, an extremely rare location; we also describe the endoscopic approach that was used to biopsy the tumor.

## Introduction

Gastrointestinal stromal tumors (GISTs), even though rare, are the most common mesenchymal tumors of the gastrointestinal (GI) tract [1]. The worldwide annual incidence rate of GISTs is approximately 1.5 per 100,000 persons. They occur most frequently in adults aged 50-70 years, and they affect both genders equally [2]. GISTs were previously classified as GI leiomyosarcomas but are now considered a distinct tumor entity within soft tissue sarcomas [3]. Most GISTs arise from the stomach (50-70%) and small intestine (20-30%), but a small percentage also occurs in the large intestine (5%) and esophagus (2-5%) [4]. In 1998, Kindblom et al. demonstrated that the cell of origin of these tumors is a pluripotent mesenchymal stem cell that is programmed to differentiate into interstitial cells of Cajal, which control peristalsis [5]. GISTs account for approximately 1-3% of all GI tumors and are known for their wide variability in biological behavior and their unpredictable malignant potential [6,7]. When GISTs occur outside of the GI tract, they are termed extragastrointestinal stromal tumors (EGISTs). Despite their location, EGISTs still maintain histopathological and molecular characteristics similar to GISTs [8]. There have been reports of EGISTs occurring in the abdominal wall, mesentery and retroperitoneum, lesser omentum, pancreas, and scrotum [9]. However, the precise incidence and tumor behavior of EGISTs is not clearly understood [10]. In this report, we present a case of EGIST that originated in the anterior perirectal space, which is an extremely rare location, and we describe the endoscopic approach that was employed to biopsy the tumor. Based on a literature review, we also discuss the incidence, pathogenesis, diagnosis, and treatment of this neoplastic disease.

## Case presentation

**Figure 1:**
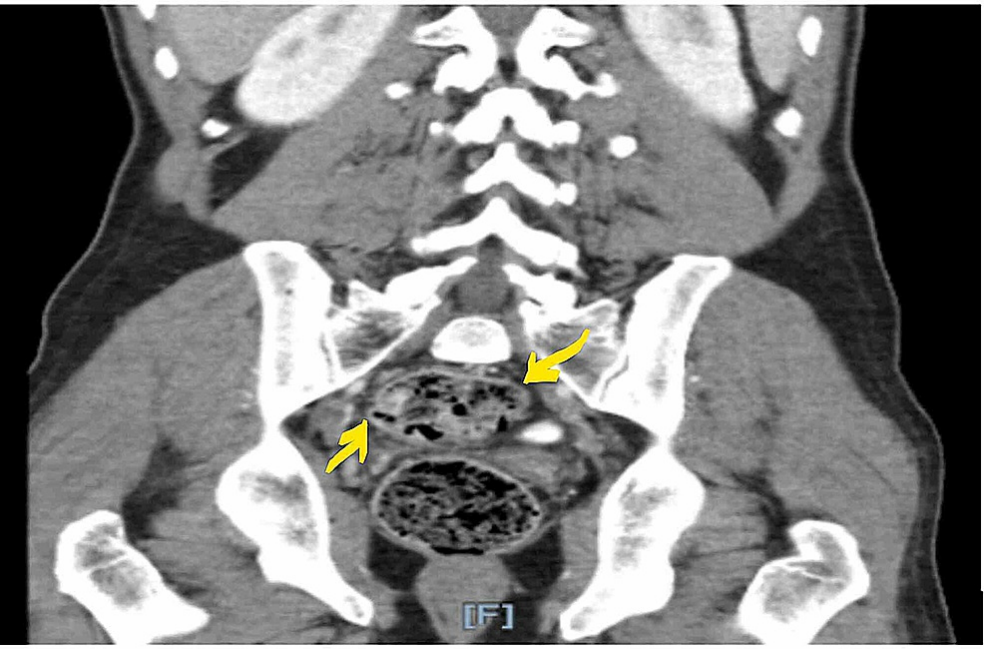
CT abdomen and pelvis CT image in the coronal plane showing a multilobular solid and cystic lobular mass in the right pelvis measuring 5.9 x 4.4 x 5.6 cm (yellow arrows), abutting the posterior dome of the bladder CT: computed tomography

**Figure 2:**
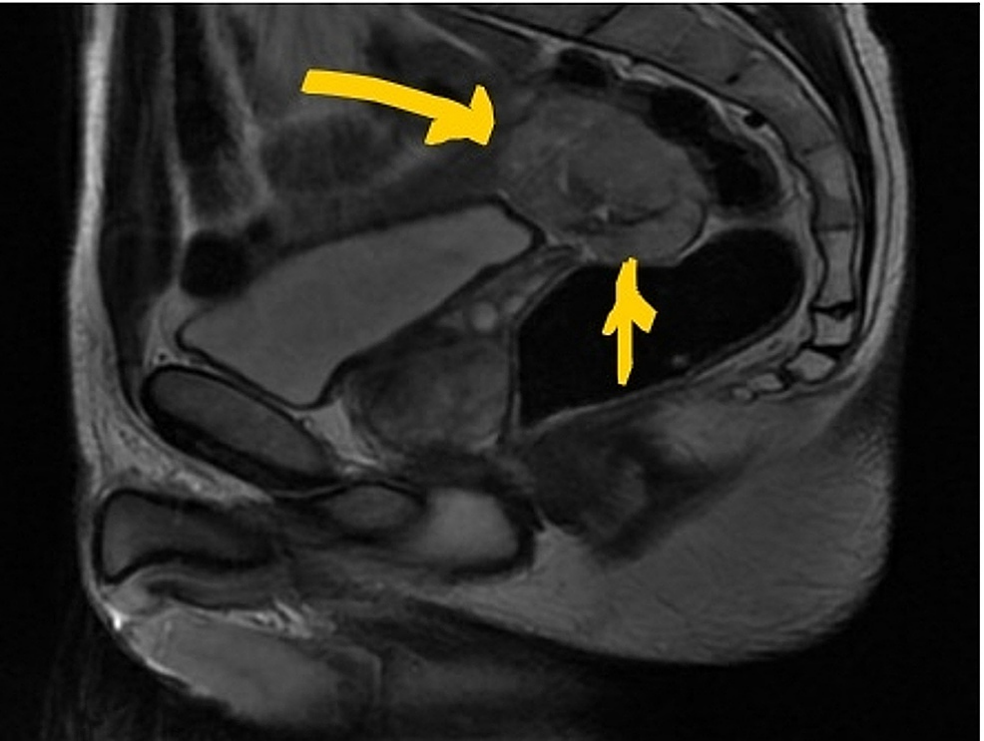
MRI image in the sagittal plane The image shows a soft tissue mass in the right pelvis, measuring approximately 7 cm. It is proximal to the urinary bladder and seminal vesicles and is unrelated to them (yellow arrows). It has heterogeneous enhancement. It is abutting the colon but does not involve the colon MRI: magnetic resonance imaging

A 41-year-old Algerian male with a past medical history of Graves' disease and vitiligo presented to the Emergency Department with right lower abdominal pain. The pain had started two weeks prior to the presentation, and it was stabbing in quality, non-radiating, and rated 6/10 in intensity, with no relieving or exacerbating factors. The patient denied any other GI or urinary symptoms and denied changes in weight or appetite. On presentation, his vital signs were stable; laboratory workup including complete metabolic panel, complete blood count, and urinalysis was unremarkable. CT scan of the abdomen and pelvis showed a multi-lobular solid and cystic mass in the right pelvis measuring 5.9 x 4.4 x 5.6 cm, abutting the posterior dome of the bladder (Figure 1). A follow-up MRI study was done for further assessment of the mass. which showed that the mass’s single largest dimension was 7 cm. It was located in the right pelvic region, abutting the sigmoid colon, but did not arise from the wall of the large intestine, and raised high suspicion for a neoplasm (Figure 2).

A multidisciplinary discussion resulted in the decision to obtain a fine-needle aspiration (FNA) of the mass guided by endoscopic ultrasound (EUS). This endoscopic approach utilized an endosonoscope, which was introduced through the anus and advanced to the rectosigmoid junction. The ultrasound images showed a heterogenous extraluminal mass found in the anterior perirectal space, which had endosonographic borders that were well defined, a maximum length of 55 mm, and a maximum width of 40 mm. There was no sonographic evidence of invasion into the rectum or urinary bladder. A few abnormal lymph nodes were visualized in the vicinity of the mass. The nodes were sub-centimeter, round, and hypoechoic with well-defined endosonographic borders. A 19-gauge core biopsy needle was passed through the rectal wall (transrectal approach), and FNA of the perirectal mass was performed. Color Doppler imaging was utilized prior to needle puncture to confirm a lack of significant vascular structures within the needle path. Two passes were made with the needle while utilizing a stylet. A cytotechnologist was present during the procedure and confirmed that the cellularity of the specimen was adequate.

**Figure 3:**
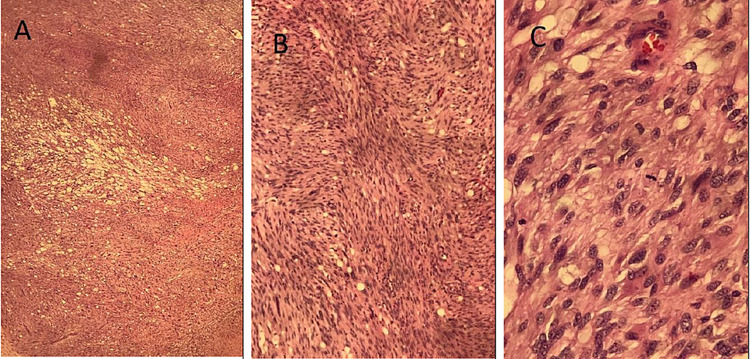
Histopathologic images in low power (A), intermediate power (B), and high power (C) The images show proliferation of spindle cells, with focal prominent cytoplasmic perinuclear vacuoles and focal marked nuclear atypia, and infrequent mitotic activity

The pathology study showed proliferation of spindle cells, with focal prominent cytoplasmic perinuclear vacuoles, focal marked nuclear atypia, and infrequent mitotic activities (Figure 3). On immunostains, the tumor cells were positive for CD117 and CD34 and negative for S100 and desmin. A stain with Ki-67 showed a low proliferation fraction (~5%). These results supported a diagnosis of GIST. A molecular genetics study was performed to guide the choice of chemotherapy. The study showed the presence of KIT (c-KIT) mutation and the absence of platelet-derived growth factor (PDGFR) mutation. 

Subsequently, the patient underwent exploratory laparotomy, which showed a pelvic mass abutting the mid jejunum and rectum. The tumor was resected and sent for histopathologic analysis, which confirmed the diagnosis of GIST. The mass had a size of 7 cm (single largest dimension) and a mitosis rate of 5 out of 50 high power fields (HPF). Using the National Institute of Health-Fletcher criteria for GIST risk assessment, we determined that this was a high-risk tumor (Table 1) [11]. The treatment plan consisted of imatinib 400 mg daily for at least two years along with periodic outpatient follow-up. The patient was hospitalized for a total of five days following the surgery and had no postoperative complications. The patient was seen in the outpatient clinic one month following his discharge from the hospital and reported no adverse outcomes. In addition, a follow-up CT study of the abdomen and pelvis was done four months after his discharge from the hospital, and the results showed no acute abdominal or pelvic pathology.

**Table 1:** Tumor risk assessment: high-risk tumor* *Refer to the National Institute of Health-Fletcher criteria for GIST risk assessment [11] HPF: high power field; GIST: gastrointestinal stromal tumor

Tumor location	Tumor size	Mitotic rate	Stage	CD	Mutations	Tumor risk
Non-gastric	7 cm	5/50 HPF	T3N1M0	117, 34	c-KIT+	High

## Discussion

EGISTs have no specific symptoms on presentation and no typical radiologic features. The most frequent associated complaint is abdominal discomfort, which may or may not be accompanied by GI bleeding [7]. This nonspecific clinical presentation hinders clinicians from making the diagnosis preoperatively. Also, the differential diagnosis tends to be broad and includes leiomyosarcoma, liposarcoma, fibrosarcoma, or solitary fibrous tumor [11]. In this case report, the EGIST presented as a heterogeneous mass in the anterior perirectal space.

The term EGIST was first used in the year 2000 by Reith et al. [12]. EGISTs are rare entities and account for less than 1% of all GI tumors. They arise from Cajal-like cells or from pluripotent stem cells outside of the GI tract, and they tend to be more aggressive and generally have a poor prognosis [5]. However, EGISTs still maintain histopathological and molecular characteristics similar to GISTs [7]. The one-year and five-year overall survival rate for EGIST is 91.7% and 48.9%, respectively, while GIST has a one-year overall survival rate of 94% and a five-year overall survival rate of 82.4% [11].

The majority of malignant GISTs have activating mutations of the c-kit gene that causes ligand-independent phosphorylation and activation of the KIT receptor tyrosine kinase, which in turn leads to tumorigenesis. Approximately 5-10% of GISTs will have mutations in the platelet-derived growth factor alpha (PDGFRA). GISTs that are wild-type for both KIT and PDGFRA mutations may show mutations in succinate dehydrogenase (SDH) B, C, or D and may be driven by the IGF-I pathway [3].

According to the National Comprehensive Cancer Network (NCCN), the workup for GIST/EGIST should include patient history, physical examination, complete blood count, liver function tests, and appropriate imaging studies such as abdominal and pelvic CT scan with contrast and/or MRI as well as surgical assessment. In selected cases, endoscopy and endoscopic ultrasound may be helpful. A biopsy is necessary to confirm the diagnosis. An endoscopic transmural biopsy can be considered if the risk of peritoneal seeding by the tumor is low. Otherwise, a percutaneous transperitoneal biopsy is more appropriate. Morphologic diagnosis based on microscopic examination of histological sections is the standard method for GIST diagnosis [13].

Over 90% of GISTs show overexpression of the receptor tyrosine kinase KIT (CD117) by immunohistochemistry (IHC), and about 5% are CD117-negative. GIST is also usually associated with CD34 expression in 70% of cases [10]. Their malignant potential is evaluated using several parameters that include size, location, invasion of adjacent organs, mucosal invasion, cellular architecture, degree of cellularity, mitotic count, nuclear pleomorphism, necrosis, and proliferation [1]. Table 1 outlines the malignant potential for the tumor presented in this case report.

The NCCN recommends the use of imatinib 400 mg in advanced or metastatic GIST patients. It also recommends augmenting the dose to 800 mg if exon 9 KIT mutation is positive. Surgical excision is the only potential cure for GIST and is aimed at resecting the tumor with histologically negative margins [14].

## Conclusions

The EGIST that we encountered in this case presented in the anterior perirectal space and was biopsied endoscopically with FNA prior to surgical resection. This endoscopic approach facilitated a timely diagnosis and treatment of a high-risk tumor. EGISTs have no specific symptoms or radiological features and can present in variable anatomic locations. They also tend to behave more aggressively and have a poor prognosis. The diagnosis of EGISTs, therefore, can be challenging and necessitates a multidisciplinary approach for accurate decision-making with regard to the biopsy method and the treatment plan. It is of paramount importance to include EGIST in the differential diagnosis when a patient presents with a neoplasm in the abdomen or pelvis. An endoscopic biopsy can be considered if the risk of peritoneal seeding by the tumor is low, and surgical excision is the mainstay of therapy. Finally, more studies with long-term follow-ups are necessary to gain deeper insights into the behavior of this rare neoplastic disease.
